# Effects of Total Enteral Nutrition on Early Growth, Immunity, and Neuronal Development of Preterm Infants

**DOI:** 10.3390/nu13082755

**Published:** 2021-08-11

**Authors:** Zakir Hossain, Wafaa A Qasem, James K. Friel, Abdelwahab Omri

**Affiliations:** 1 Department of Fisheries Biology and Genetics, Bangladesh Agricultural University, Mymensingh 2202, Bangladesh; 2 Department of Surgery, Mubarak AlKabeer Hospital, Hawally 32052, Kuwait; Wafaa.qasem@hsc.edu.kw; 3 Community Medicine Department, Faculty of Medicine, Kuwait University, Kuwait City 13003, Kuwait; 4 Richardson Centre for Functional Foods and Nutraceuticals, Department of Human Nutritional Sciences, University of Manitoba, Winnipeg, MB R3T 6C5, Canada; james.friel@umanitoba.ca; 5 The Novel Drug and Vaccine Delivery Systems Facility, Department of Chemistry and Biochemistry, Laurentian University, Sudbury, ON P3E 2C6, Canada; aomri@laurentian.ca

**Keywords:** preterm infants, human milk, macronutrients, minerals, vitamins

## Abstract

The feeding of colostrum and mother’s transitional milk improves immune protection and neurodevelopmental outcomes. It also helps with gut maturation and decreases the risks of infection. The supply of nutrients from human milk (HM) is not adequate for preterm infants, even though preterm mother’s milk contains higher concentrations of protein, sodium, zinc, and calcium than mature HM. The human milk fortifiers, particularly those with protein, calcium, and phosphate, should be used to supplement HM to meet the necessities of preterm infants. The management of fluid and electrolytes is a challenging aspect of neonatal care of preterm infants. Trace minerals such as iron, zinc, copper, iodine, manganese, molybdenum, selenium, chromium, and fluoride are considered essential for preterm infants. Vitamins such as A, D, E, and K play an important role in the prevention of morbidities, such as bronchopulmonary dysplasia, retinopathy of prematurity, and intraventricular hemorrhage. Therefore, supplementation of HM with required nutrients is recommended for all preterm infants.

## 1. Introduction

In developed countries, demographic factors such as maternal age, rates of twinning, and assisted conception are increasing daily; as a result, the delivery rate of preterm infants is increasing daily. The survival of preterm infants continues to increase in both developed and developing countries, which, in part, reflects improvements in nutritional care. The internationally accepted guidelines for enteral nutrition produced in 2010 have been widely adopted, with many aspects having been reviewed again more recently [1,2]. The deficiency of basic food components such as protein, fat, carbohydrate, minerals, and vitamins in the early life of preterm infants undesirably affects the growth and functions of vital organ systems. Therefore, all the essential nutrient components need to be supplied to preterm infants in good time. A number of articles have been published on the nutrition needs of preterm infants; most of these articles highlight a single nutrient. None of the published articles focused on the total nutrition of preterm infants. This review article focuses on the growth, immunological, and neurodevelopmental effects of total enteral nutrition in preterm infants.

## 2. Oropharyngeal Colostrum 

The colostrum of preterm infant’s mothers is rich in immune-protective components compared with mature breast milk [3,4,5]. Initially, exposure to colostrum may not occur because feedings are often withheld due to irregular gastric motility. In some cases, preterm infants have delayed feedings, and the administration of intragastric tube feedings may be difficult because of their vulnerable health condition [6,7,8]. The maturation of immunity in the preterm infant is related to increases in the mother’s milk production and colostrum feeding [9]. Colostrum also has a role in the microbiome, which is enhanced lymphoid tissue, bacterial adhesion, and alterations to bacterial colonization in the trachea, and provides immunoglobulin A (sIgA) to the preterm infants [10,11]. The sIgA inhibits pathogens growing on the mucosal surface, and lactoferrin plays a role in anti-inflammation; as a result, the mucosal barrier receives high levels of protection. Colostrum antioxidants work as a safeguard for preterm infants by protecting the mucosal damage caused by free radicals and conserving the healthy membrane, and colostrum biofactors protect from necrotizing enterocolitis (NEC) [12,13]. Colostrum may protect preterm infants from bronchopulmonary dysplasia and sepsis [14]. 

## 3. Human Milk

Human milk (HM) contains hormonal and enzymatic components, anti-infective, trophic and growth factors, stem cells, prebiotics and probiotics, and a myriad of bioactive proteins, which make it the gold standard for both full term and preterm infants [15,16,17,18,19,20]. Preterm infants miss the nutrition from their mother in the third trimester; as a result, mother’s milk alone is not sufficient to provide adequate nutrition. Therefore, it is essential to supplement mother’s milk with additional nutrients for the fulfillment of nutrition requirements to the preterm infants [21]. The supplementation with protein, carbohydrate, fat, minerals, and vitamins with HM is needed to fulfil the nutritional needs of preterm infants [22]. Preterm formula that contains required nutritional elements is a suitable option for preterm infants. Abramovich et al. (2013) found that the nutrients in stored (−80 °C) human milk were not changed, except vitamin C, until 6 months [23]. Therefore, in cases where the own mother’s milk is unavailable, storage milk from the milk bank can be used for preterm infants; it is rich in fat, lactose, and vitamins and produced during the 3rd–14th day postpartum, while transitional milk is followed by mature milk. The milk of mothers who delivered preterm infants contains higher levels of protein, sodium, chloride, calcium, zinc, copper, and folate than full-term HM [24]. HM protects preterm infants from NEC and sepsis [25,26], retinopathy of prematurity (ROP) [27,28], bronchopulmonary dysplasia [29], and neurocognitive and cardiovascular problems [30,31].

## 4. Human Milk Fortification 

Human milk fortifiers (HMFs) contain several macronutrients as well as minerals—calcium, phosphorus, sodium, and zinc—and vitamins—A, D, E, K, riboflavin, folic acid, thiamine, pyridoxine, and nicotinamide. HMFs are available in powdered or liquid form; powdered fortifiers should be mixed in HM thoroughly considering the optimum osmolality for better absorption. Pragmatic methods were used to express requirements, including those for nutrients such as minerals [32,33] (Table 1). However, the requirements are dependent on the gestation age and the clinical condition of preterm infants; therefore, HMFs should be added on the basis of the specific needs of preterm infants. Some HMFs use human milk, but most use bovine or donkey milk. HMFs come as multi-nutrient fortifiers or single-nutrient fortifiers. Recently, donkey milk has been used in HMFs, as it is very close to HM [34].

Multi-nutrient HMFs contain different amounts of protein, carbohydrate, fat, minerals, trace-elements, and vitamins; the osmolality of the HMFs was reduced by decreasing the carbohydrate content [35]. The lipids in HMFs improve the status of essential fatty acids (EFA) in preterm infants [36]. HMFs with higher protein content increase preterm infants’ weight gain [37]. Diehl-Jones et al. (2015) suggested the optimization of HMFs based on their cellular and genomic study; this minimizes the production of harmful free radicals in the very-low birthweight (VLBW) preterm infant, who is at great risk of prematurity diseases [38]. Single-nutrient supplements are useful during individualizing fortification such as dextrin maltose and medium-chain triglycerides, which are used as supplements of carbohydrate and lipids, respectively; recently, an HM-derived cream supplement has been introduced to increase the energy of feeds [39,40,41,42]. A partially hydrolyzed protein is designed to be added to the HMFs [35]. Hossain et al. (2014) reported that HMF supplementation can be optimized to promote the growth of the preterm infant. The optimal osmolarity should be maintained, as high osmolality could cause prematurity diseases. Preterm infants fed HM + HMF showed higher lipid oxidation; in addition, HM + HMF upregulates antioxidant genes in cultured fetal intestinal cells. The HMFs can serve the nutritional requirements of the preterm infants if we can optimally provide HMFs to the individual [43].

## 5. Protein

Preterm infants take in protein in different forms such as amino acids (AA) in parenteral nutrition, high-protein preterm formulas, and the supplementation of HM with liquid protein [44,45,46,47]. The supplementary protein, particularly in VLBW preterm infants, improves growth, including in essential regions in the brain related to cognitive outcomes [48]. Slower growth rates and poorer cognitive function are the result of the under nutrition of preterm infants [49]. AA and protein are crucial for normal fetal growth and development, providing support for the growth of all cells, tissues, and organs by regulating the metabolism. Leucine and arginine particularly stimulate insulin secretion, which enhances protein synthesis and protein accretion [50]. Leucine and arginine are the vital oxidative substrate [51] and basic substrate for nitric oxide [52], respectively; these play important roles in vascular function and blood flow to growing organs. Additionally, glutamate, serine, and other non-essential amino acids exclusively promote placental and fetal metabolism [53,54]. The supply of AA has roles in fetus growth and cognitive development [54].

## 6. Fat

Energy is used as a fuel during tissue synthesis. Dietary fat is the primary source of energy for preterm infants; the oxidation of fat provides energy to support basal metabolic functions [55]. The fat requirements are based on the amount of energy needed for suitable growth and the amount of ω3 and ω6 polyunsaturated fatty acids needed for the functioning of the cell membrane, eicosanoid metabolism, and development central nervous system [56]. Hossain et al. (2016) suggested that metabolites of fatty acids from food sources of preterm infants are reflected in the circulating plasma fatty acids [57]. The lipid metabolizing enzymes, lipases, and carnitine palmitoyltransferase are at low levels in VLBW preterm infants. Lipase clears the plasma triglycerides, and carnitine palmitoyltransferase transports long chain fatty acids into mitochondria for energy production through the oxidation of lipids. Since HM contains lipase and carnitine, enterally feeding HM immediately after a preterm infants’ birth is the best strategy to stimulate lipid oxidation and energy production. In addition, formulas have more 22:6n-3 (DHA); increased DHA-fed preterm infants had increased DHA levels and showed higher visual acuity, improved Bayley mental development, and better MacArthur communicative inventories [58]. 

## 7. Carbohydrate

Carbohydrate is an important source of energy for the growth and development of preterm infants; HM and many formulas contain lactose as a carbohydrate source [59]. Maltodextrin has helped to reduce the osmolarity and related intestinal distress [60]. On the other hand, glucose is a major energy source for perinatal brain development under normal physiological conditions [61]. High glucose levels increase the risks of hyperglycemia, including reduced insulin secretion, and limit the enteral feedings; hyperglycemia is associated with an increased incidence and severity of ROP [62]. It also increases the production of reactive oxygen species, which could cause cellular damage and cell death as well as inflammation [63,64]. To avoid detrimental effects on outcomes in preterm infants, the optimal composition of early PN to avoid postnatal growth failure must be carefully balanced against hyperglycemia risk; hypeglycemia increases the risk of long-term neurodevelopmental delay in surviving preterm newborns [65]. Bacteria flourish in high-glucose environments, and hyperglycemia leads to dysfunctional intestinal tight junctions; as a result, tight junction permeability increases, and finally, microorganisms may reach the circulatory systems, which might lead to bacterial sepsis and NEC [66]. The administration of insulin in preterm infants will lower plasma glucose levels; however, there are several risks, causing significant concern [67]. 

## 8. Mineral Requirements for Preterm Infants

Minerals such as iron, calcium, phosphorus, magnesium, potassium, and sodium are needed for the proper growth and development of preterm infants. Among their other physiological roles in humans, calcium, phosphorus, and magnesium are essential for bone development [68], and potassium and sodium play a role in water fluxes and enzymatic functions [69], while iron is essential for optimal brain development [70].

### 8.1. Calcium and Phosphorus 

An insufficient supply of calcium (Ca) and phosphorus (P) is a major reason for weakened bone development in preterm infants [71]. In the full-term infant, up to 80% of the body Ca is grown during the last trimester [72]. Since preterm infants miss the last trimester, they need mineral supplementation. It is challenging to meet the Ca and P requirements in preterm infants, as the solubility of Ca and P in parenteral fluid has a limit, the HM has a low Ca and P content, and intestinal absorption of minerals during formula feeding is uneven [73,74]. Therefore, Ca and P supplementation is essential to prevent impaired bone mineralization. Therefore, HMFs with HM are the best option to provide Ca and P supplementation [75]. 

### 8.2. Magnesium

Magnesium (Mg) plays a crucial role in bone matrix growth and the synthesis of biomacromolecules, including DNA, RNA, proteins, and glycolysis [76]. Electrolytes and minerals are provided to preterm infants through the administration of Mg in parenteral nutrition [68]. The fetal need for Mg occurs throughout pregnancy; 3 to 5 mg/kg/day is necessary during the third trimester. The need for Mg increases with increased gestation age [77,78]. Therefore, Mg supplementation is essential for preterm infants, since they miss the in utero use of Mg during the third trimester.

### 8.3. Sodium

Sodium (Na) plays a vital role in the growth, DNA synthesis, cell proliferation, and absorption of nutrients in preterm infants [78,79]. The recommended daily Na intake in healthy infants is 2 to 3 mEq/kg/d, compared with 3 to 5 mEq/kg/d in preterm infants [80]. Kidney function in preterm infants is usually established by 32 to 34 weeks of gestation age; the distal tubule and loop of Henle are responsible for sodium excretion and reabsorption [81]. Preterm infants need to absorb and excrete Na under inadequate and excess Na conditions [82]. Therefore, Na is essential in preterm infants to reduce the risk for Na deficiency.

### 8.4. Potassium 

Potassium (K) plays an important role in protein synthesis, cell growth, and cell volume regulation. Preterm infants with a birth weight of 1 < kg and gestation age of <30 weeks showed 6 meq/L mean plasma K^+^ in the first day of life; the K^+^ stabilizes between days 4 and 5 [83]. It is important to keep the reference values of K^+^, as hyperkalemia can cause serious complications in preterm infants. The value of plasma K^+^ in preterm infants is elevated compared to older infants, children, and adults.

### 8.5. Iron

Iron deficiency affects the growth and functions of multiple organ systems in preterm infants [84]. Preterm infants miss the iron deposition from their mother during the third trimester [85]. Therefore, preterm infants are at high risk of iron deficiency, and the supplementation of iron is essential for their normal growth and an early inception of erythropoiesis [86]. During iron supplementation, the dose should be optimized, as excess iron produces circulating Fe2þ ions, which can lead to the production of reactive oxygen species that can affect the heart, liver, pancreas, and developing brain functions [87]. To achieve the optimum dose, international consumption recommendations for enteral iron in preterm infants are 2–3 mg/kg/d from the age of 2–6 weeks until at least 6–12 months [2,88]. Qasem et al. (2017) found that iron-fortified complementary feeding developed the infant’s gut microbiota [89]. Iron-fortified HMF may develop the gut micribiota of preterm infants.

## 9. Preterm Trace Mineral Requirements 

Trace minerals such as iron, zinc, copper, iodine, manganese, molybdenum, selenium, and chromium are essential, and humans need to receive them in diet [90]. The role of selected trace minerals, symptoms of deficiency, and ASPEN and ESPHAN recommendations for trace mineral intake in preterm infants are summarized in Table 2 [91,92,93]. HM is the “gold standard” for trace mineral content for the healthy full-term infant, while no gold standard exists for preterm infants. Therefore, the supplementation of trace mineral intakes is essential through parenteral and enteral nutrition in preterm infants [90,94]. 

### 9.1. Zinc

Zinc (Zn) is a crucial factor in growth and cell differentiation and the metabolism of proteins, carbohydrates, and lipids; it also plays a vital role in hormone structure, metalloenzymes, the maturation of intestine, immune function, and genetic transcription factors [90,95]. Preterm infants need Zn supplementation due to the insufficient accretion of Zn stores, and excretion through an immature intestinal tract [96]. Two-thirds of the total body Zn is transferred from maternal stores to full-term infants during third trimester, while preterm infants are susceptible to Zn deficiency if Zn supplementation is not available early. A double-blind, randomized controlled study showed significantly lower morbidity and mortality in the VLBW preterm infants supplemented at a dose of 10 mg/d Zn at postnatal day 7 compared with the control group [97]. Preterm neonates may have higher parenteral requirements for zinc than previously recommended during the first week of life [98]. Zinc supplementation for >2 weeks improved fronto-occipital circumference growth but not linear growth or weight gain [99].

### 9.2. Copper

Copper (Cu) is a component of numerous metalloenzymes and thus plays a vital role in metabolic processes. The superoxide dismutases and ceruloplasmin protect cell membranes from oxidative damage and work as the major Cu transporter in plasma, respectively [100]. Preterm infants have lower plasma Cu levels at birth, with mean serum Cu levels ranging from 20 to 50 mcg/dL at 1 week of postnatal age [90]. The Cu requirements in VLBW preterm infants are higher than for full-term infants; in addition, hepatic Cu in VLBW preterm infants is lower than in full-term infants. The symptoms of Cu deficiency are hypochromic anemia resistant to iron supplementation, pancytopenia, poor wound healing, and a variety of bone abnormalities [97,101]. Erythrocyte superoxide dismutase and cytochrome oxidase are important bio-indicators of Cu status in the preterm infant [101]. 

### 9.3. Selenium

Selenium (Se) works on antioxidant defense through glutathione peroxidase, which participates in antioxidant defense and helps scavenge free radicals to protect the body from oxidative damage [102]. The intrauterine accretion of Se stores mainly occurs in the third trimester in preterm infants, as Se concentrations in the plasma are lower compared to full-term infants [103]. Makhoul et al. [104] also found that Se concentrations increase with increased gestation age after 36 weeks of gestation. A randomized double-blind study on VLBW preterm infants receiving Se supplementations at concentrations of 7 mcg/kg/d parenterally and 5 mcg/kg/d with HM or formula or no additional supplementation showed that Se concentrations were significantly lower in the no supplementation group [105]. The symptoms of Se deficiency are myocardial disorders, skeletal muscle disorders, erythrocyte macrocytosis, fingernail-bed abnormalities, and pseudoalbinism [106]. 

### 9.4. Manganese 

Manganese (Mn) plays important roles in manganese-dependent superoxide dismutase and pyruvate carboxylase and other enzyme systems [90]. Mn is found in a high concentration particularly in the liver and brain even though it is found in all tissues of the body. Mn deficiency affects mucopolysaccharide and lipopolysaccharide formation, impaired skeletal development, and ataxia [90,91]. Liver cholestasis, insomnia, hyperbilirubinemia, headache, anxiety, rapid hand movements, cerebral and hepatic complications, and Parkinson disease are the symptoms of excessive intake of Mn in PN [107,108,109]. 

### 9.5. Chromium, Molybdenum, and Iodine 

Chromium (Cr) plays vital roles in protein, carbohydrate, and lipid metabolism. The symptoms of Cr deficiency are weight loss, high-plasma free fatty acid concentrations, and glucose intolerance. Cr supplementation in parenteral nutrition is recommended in preterm infants for 4 weeks [94]. A randomized controlled study showed that Cr intake for 3 weeks significantly increased the creatinine values in preterm infants [110]. Molybdenum (Mo) is essential for enzymatic systems such as xanthine dehydrogenase/oxidase, aldehyde oxidase, and sulfite oxidase [111]. The deficiency symptoms are tachycardia and coma and high levels of sulfite and urate in the blood. It was found that 1 mcg/kg/d of parenteral Mo and 4–6 mcg/kg/d of enteral Mo were adequate for LBW preterm infants [112]. Iodine (I) is a component of the thyroid hormones triiodothyronine (T3) and thyroxine (T4), which regulate protein metabolism. Preterm infant formulas, own mother’s milk, and donor human milk contain 20–170, 50–150, and 33–117.5 mcg/L iodine, respectively [113,114]. Studies of balance suggest that iodine intakes of ≥30 mcg/kg/d are required for preterm infants to maintain a positive balance. A parenteral intake of 1 mcg/kg/d was recommended to avoid the risk of iodine deficiency in preterm infants [92]. 

## 10. Recommendations Concerning Vitamins

### 10.1. Fat-Soluble Vitamins

#### Vitamin D 

The deficiency of vitamin D in preterm infants is common, as infants have missed the opportunity to store vitamin D from the mother during the third trimester, lacked sunlight exposure during the sick period, and face difficulties in adequate enteral nutrition. The supplementation of vitamin D is necessary to provide a sufficient vitamin D level to preterm infants; the daily vitamin D supplementation recommendation is from 800 to 1000 and 400 IU/day for preterm infants in Europe and western countries, respectively [115]. 

#### Vitamin A

ROP is a common visual disorder in preterm infants, associated with the regulation of the vascular endothelial growth factor and insulin-like growth factor [116,117]. Vitamin A deficiency is prominent in VLBW preterm infants, as infants have missed the opportunity to store it from their mothers during the third trimester, receive an insufficient intake from enteral feeding, and have poor gastrointestinal absorption [118]; hepatic storage of vitamin A is not as efficient in extremely preterm infants [119]. The intramuscular supplementation of vitamin A may prevent the ROP [120], and supplementing the feeds with 5000 IU of vitamin A per day could mitigate the vitamin A deficiency; it was associated with a decreased incidence of Type 1 retinopathy of prematurity and may also have a positive impact on reducing bronchopulmonary dysplasia [121,122].

#### Vitamin E

Vitamin E works as a free radical scavenger [123,124], which can prevent bronchopulmonary dysplasia, ROP, and intra-ventricular hemorrhage [123]. The American Academy of Pediatrics suggests supplementing vitamin E in all preterm formulas and parenteral nutrition for preterm infants [125]. In addition, Kositamongkol et al. [126] found a deficiency in 77.4, 16.1, and 35.7% of 35 VLBW preterm infants at birth, at the postnatal age needed to reach full feedings and at term postconceptional age, respectively. 

#### Vitamin K 

Vitamin K is essential for preterm infants, where deficiency may kill or cause a permanent, serious handicap [127]. The activity of hepatic vitamin K1 2, 3-epoxide reductase is low in preterm infants; as a result, less efficient recycling of vitamin K is observed in preterm infants [128]. HM contains very low concentrations of vitamin K1 [127]; therefore, HM-fed preterm infants need vitamin K supplementation to avoid the risk of deficiency [129].

## 11. Conclusions

Preterm infants have insufficient nutrient stores at birth, and nutrient supplementation is essential for the growth and functions of vital organs. This review has highlighted the need of total enteral nutrition for preterm infants to improve early growth, immune status, and neuronal effects. In addition, based on this review, we suggest that the following clinical practice points are considered:

1. Oropharyngeal colostrum administration appears to be safe and promotes immunological maturation;
2. HM can provide many essential elements for preterm infants; if it is not available, donor HM can be provided, even if it is not an equivalent to the own mother’s milk;
3. Earlier feeding initiation, human milk fortification, and certain formulas appear to improve the growth and neurodevelopmental outcomes;
4. Carbohydrate, protein, fat, minerals, trace minerals, and vitamins should be provided through PN or EN considering the requirements of preterm infants for their better growth and development.

## Figures and Tables

**Table 1:** Requirements for protein and energy; best estimates by factorial and empirical methods (33).

Body Weight (g)	500–1000	1001–1500	1501–2000
Weight gain of fetus (g/kg/day)	19.0	17.4	16.4
Protein gain of fetus (g/kg/day)	4.0	3.9	3.7
Energy (Kcal/kg/day)	106	115	123
Protein/energy (g/100 kcal)	3.8	3.4	3.0

**Table 2:** Role, deficiency symptoms, and ASPEN and ESPHAN recommended dose of trace minerals [90,91,92].

Trace Minerals	Role	Deficiency Symptoms	ASPEN Recommended Dose for Preterm Infant (µg/kg/day)	ESPHAN Recommended Dose for Preterm Infant (µg/kg/day)
Zinc	Cofactor for >300 metalloenzymes; important for growth, cell differentiation, and the metabolism of proteins, carbohydrates, and lipids; plays a role in hormone structure, gastrointestinal (GI) development, immune function, and genetic transcription factors	Poor growth; weight loss; periorificial dermatitis; glossitis; increased susceptibility to infections; diarrhea	300	400–500
Copper	Free radical scavengers-help protect cell membranes from oxidative damage; essential to the proper functioning of organs and metabolic processes	Hypochromic anemia resistant to iron; pancytopenia; poor wound healing; osteopenia; fractures	20	40
Selenium	Free radical scavenger; antioxidant; can reduce the risk of sepsis; plays a role in thyroid hormone metabolism	Myocardial disorders; skeletal muscle disorders; erythrocyte macrocytosis; fingernail-bed abnormalities; pseudoalbinism; growth retardation; alopecia	2	7
Manganese	Cofactor for several enzymes, including superoxide dismutase and pyruvate carboxylase	Affects mucopolysaccharide and lipopolysaccharide formation; impaired skeletal development and ataxia (animal models)	1	≤1
Chromium	Important for macronutrient metabolism; enhances the action of insulin	Insulin-resistant hyperglycemia; glucose intolerance; weight loss; high plasma free fatty acid concentrations	0.0006	0
Molybdenum	Required by 3 enzymatic systems: xanthine dehydrogenase/oxidase, aldehyde oxidase, and sulfite oxidase	Cardiac and neurologic symptoms, including tachycardia and coma; high blood levels of sulfite and urate	NG	1
Iodine	Major component of thyroid hormones that regulate key biochemical reactions in energy and protein metabolism; necessary for growth, development, and maturation	Hypothyroidism; poor growth; poor neurodevelopment; cretinism; goiter	NG	1–10

## Data Availability

Upon request data will be available.
